# Assessment of sow herd frequency of PCV-2 using placental umbilical cord serum and serology in 18 breeding farms in Brazil

**DOI:** 10.3389/fvets.2024.1368644

**Published:** 2024-04-08

**Authors:** Ricardo T. Lippke, Elisa R. De Conti, Luciana F. Hernig, Angélica P. Teixeira, Fernando A. de Quadros, Aparecida T. Fiúza, Juliana B. Pereira, Rafael da Rosa Ulguim, David E. S. N. Barcellos, Karine Ludwig Takeuti

**Affiliations:** ^1^Boehringer-Ingelheim Animal Health do Brasil, São Paulo, Brazil; ^2^Setor de Suínos, Universidade Federal do Rio Grande do Sul, Porto Alegre, Brazil; ^3^Universidade Feevale, Novo Hamburgo, Brazil

**Keywords:** PCV-2, PCR, piglet viremia, serology, swine

## Abstract

Porcine circovirus type 2 (PCV-2) is the agent of one of the most important diseases in the swine industry. Although it has been controlled through vaccination, viremic piglets at birth may represent a risk by reducing vaccination efficacy. Since there are few reports on the viremic status of pre-suckling piglets regarding PCV-2 infection, we assessed the PCV-2 frequency in sows housed in 18 breeding farms with no history of clinical PCVAD in Brazil, using placental umbilical cord serum (PUCS). The selection criteria were: breeding farms with more than 1,000 sows; sows not vaccinated for PCV-2 at least for 2 years prior to the study; farms with no history of PCV-2 clinical disease in the last 12 months; and production systems with a maximum of two sites. Blood from the umbilical cords in sow placenta or directly from piglet’s immediately after birth was collected from 30 litters on each farm for PCR. In addition, blood from 538 sows was collected for PCV-2 antibody detection. A total of 17.29% of the PUCS tested positive. The PCV-2 DNA was detected in PUCS from 94.4% of all farms. A total of 94.8% of the sows was positive for PCV-2 antibodies. However, seronegative sows were detected in 44.4% of farms. All 18 farms had at least 46.9% seropositive dams. A higher percentage of seronegative sows was observed for farms with more than 10% of PCV-2-positive litters compared to those with ≤10% of PCV-2 positive litters (8.9 +/−1.7% vs. 1.5 +/− 0.7%, *p* < 0.01, respectively).

## 1 Introduction

Porcine circovirus type 2 (PCV-2) is one of the most economically important pathogens in the swine industry worldwide (1). This virus is endemic in pig farms and causes several clinical syndromes unified as “porcine circovirus-associated diseases” (PCVAD), encompassing porcine dermatitis and nephropathy syndrome (PDNS); PCV-2 lung disease (PCV2-LD); PCV-2 enteric disease (PCV2-ED); PCV-2 systemic disease (PCV-2-SD); PCV-2 reproductive disease (PCV2-RD), and PCV-2 subclinical infection (PCV2-SI) (1–4). The major differences between subclinical and clinical infections are the presence of clinical signs, losses in production, and the severity of lesions (5). Whilst subclinical PCV-2 infections are usually correlated with no gross findings and the absence or presence of mild microscopic lesions, clinical infections are characterized by moderate to severe lesions in the affected tissues (6).

The PCV2-SD is one of the most common syndromes reported in the literature and has frequently been observed in the field since 2002 (2). However, the epidemiology of PCV-2 has been modified substantially, and nowadays, PCV2-SI is the most prevalent form of manifestation due to the massive use of efficient commercial vaccines worldwide (5). According to the vaccination protocol adopted (piglets and gilts, gilts/sows only, gilts/sows and their litter vaccination, or piglets only), the PCV-2 infection dynamics probably changed in the mid-long term (5). The vaccination of gilts and sows prior to insemination is justified by evidence that piglets may become infected with PCV-2 in the uterus via transplacental infection (7–9). The progression for clinical signs of the disease in sows is dependent on a high viral replication or protracted viremia and the timing of viral infection, which can also determine the occurrence of clinical reproductive failure (10).

Additionally, piglets are frequently vaccinated at weaning to improve performance and reduce viremia, which is achieved even in the presence of maternal delivered antibodies. However, several pigs may reach the end of the finishing phase seronegative for PCV-2 if vaccination has been performed only at weaning (11, 12). In the case of multiplier farms, the absence of later vaccination could increase the chances of the emergence of subpopulations of replacement gilts with low antibody titers against PCV-2 (5). This situation can increase the prevalence of infected newborn piglets (13–16), and colostrum protection from sows to their offspring might be weak (17). A high number of pre-suckling infected piglets has been associated with a higher chance of horizontal transmission of PCV-2 among littermates, increasing the number of infected piglets at weaning. Consequently, a decrease in vaccination efficacy may be observed since the piglets would already be infected with PCV-2 at the time of vaccination. Some of them may develop the clinical disease earlier (6–8 weeks of age) or maintain a subclinical condition (PCV2-SI), with impacts on their zootechnical performance (18–20). However, these problems are not limited to piglets born to primiparous sows.

The detection of high titers of PCV-2 antibodies or the presence of seronegative sows have been reported in sows from different parity orders (13, 21). Blood sampling soon after farrowing has been used to assess PCV-2 serology in sows (10). Additionally, placental umbilical cord serum (PUCS), pre-suckling piglet serum, umbilical cord tissue, colostrum, and fetal tissues have been used to determine sow herd PCV-2 prevalence through the measure of PCV-2 viremia (8, 15, 22–25). The use of PUCS as a sample to detect PCV-2 has a high sensitivity, similar to that of colostrum and pre-suckling serum and higher to that of sow serum and processing fluids (23, 26). PUCS was used to determine prevalence of PCV-2 infection as a diagnostic specimen in some studies (16, 24) and also to evaluate the effects of sow vaccination against PCV-2 in different stages of the production cycle (22). Therefore, the aim of this study was to assess the frequency of viremic newborn piglets for PCV-2 using qPCR based on PUCS and to determine immune status of sows using serology (ELISA). Also, the relationship between seronegative sows and positive PUCS for PCV-2 in 18 breeding farms with no history of clinical PCVAD in Brazil was assessed.

## 2 Materials and methods

The Research Committee of the Federal University of Rio Grande do Sul approved the use of animals in this study (#44611).

### 2.1 Farm selection

A total of 18 farms (abbreviated from A-R) from four pig companies (X, Y, Z, and W), located in the main Brazilian pork-producing states (27), were randomly selected for the study (5 in Mato Grosso – MT, 1 in Paraná – PR, 5 in Santa Catarina – SC, and 7 in Rio Grande do Sul – RS). All farms were selected according to the following criteria: size (>1,000 sows); farms which had not vaccinated sows for PCV-2 for at least 2 years (except prepubertal gilts); farms with no history of clinical PCVAD in the last 12 months; farms with a maximum of two sites (sow farm or sow farm with nurseries) (Table 1). These farms represent the commonest piglet production models in Brazil. All farms presented a regular parity order distribution (PO – 1 to 12), except farm C, which had only primiparous sows, and farms A and B, which had no primiparous sows (PO from 2–12). The average weaning age varied from 21 ± 4 (Company X) to 23 ± 4 days (Companies Y, Z, and W). All farms were historically negative for PRRSv.

**Table 1:** Description and distribution of the farms and number of sampled animals per farm.

Farm	Company	Location	Sites	Sows/farm	Number of sow samples	Number of PUCS samples
A	X	SC	W/N	6,000	31	31
B	X	SC	W/N	4,000	30	30
C	X	SC	W	2,000	28	28
D	Y	SC	W	3,770	30	30
E	Y	SC	W	3,200	32	32
F	Z	RS	W	1,500	30	30
G	Z	RS	W	1,000	30	30
H	Z	RS	W	7,500	29	29
I	Z	RS	W	2,330	29	29
J	Z	RS	W	2,880	30	30
K	Z	RS	W/N	2,150	30	30
L	W	MT	W	3,250	30	30
M	W	MT	W	4,400	30	30
N	W	MT	W	4,400	30	30
O	W	MT	W	4,400	30	30
P	W	MT	W	4,400	30	30
Q	W	MT	W	4,400	29	29
R	W	PR	W	5,000	30	30
Total				66,580	538	538

W, breeding farms with piglets up to weaning; W/N, breeding farms with nurseries; SC, Santa Catarina; RS, Rio Grande do Sul; MT, Mato Grosso; PR, Paraná – PR.

### 2.2 Sow blood collection and placental umbilical cord serum sampling

Placental umbilical cord serum was collected directly from the placenta within 12 h post-farrowing or directly from the umbilical cord of piglets immediately after birth and before colostrum intake. Convenience sampling was performed, in which the litters from the sows that were farrowing during the time that the farms were visited were selected. A total of three to four umbilical cords per litter (29–32/farm) were stored inside sterile tubes with clot activator to reach a volume of least 3 mL. Each tube was considered one PUCS sample. To minimize sample contamination with environmental PCV-2 DNA, all utensils (e.g., scissors) were cleaned mechanically with disposable paper, washed with water and disinfected with 70% ethanol, and gloves were changed for each PUCS sampling according to Pleguezuelos et al. (22). Additionally, blood samples were collected from 29–32 sows per farm up to 4 days after farrowing, being the sows not the same as the ones from the PUCS litters. All samples (PUCS and blood from sows) were stored at 4°C for 24 h. Subsequently, the samples were centrifuged (3,000 rpm for 15 min) to obtain the serum, which was placed in 1.5 mL sterile microtubes and stored at −20°C to be tested within 30 days. A total of 1,076 samples from piglets and sows (PUCS and blood, respectively) were collected (Table 1).

### 2.3 Diagnostic test methods

#### 2.3.1 Serology

All serum samples from sows were tested for the presence of anti-PCV-2 antibodies using a commercial enzyme-linked immunosorbent assay (indirect ELISA) kit (Porcine Circovirus type 2 Antibody Test, BioChek B.V., Reeuwijk, Holland) according to the manufacturer’s instructions. Serum samples were considered positive for the presence of PCV-2 antibodies when the *S*/*p* ratio was ≥0.5.

#### 2.3.2 PCR detection for PCV-2

The DNA was extracted from PUCS using commercial kits (NewGenePrepAmp, Simbios Biotecnologia, Cachoeirinha, Brazil). The PCV-2 quantitative real-time PCR (qPCR) was performed using the NewGene PCVAmp kit (Simbios Biotecnologia, Cachoeirinha, Brazil) and specific primers (28). The standard curve was generated using references with high (10,000,000 genomes/mL), low (10,000 genomes/mL), and intermediate (10,000–1,000,000 genomes/mL) load, considering a linear regression coefficient (*R*^2^) above 0.9. The qPCR was considered positive with a Ct value <37. Viral titers inferred from the PCR results were expressed as the viral copy number per milliliter (copies/mL).

### 2.4 Statistical analysis

All statistical analyses were performed using the Statistical Analysis System – SAS^®^ 9.4 (SAS Institute Inc., Cary, NC, United States). Descriptive analyses were carried out to present the means ± standard error, quartiles, and frequencies of PCV-2 genome sequence and *S*/*p* values, considering all samples or by farm. The Pearson correlation (PROC CORR procedure) was applied using the farm as observational unit to associate the percentage of positive PUCS with the average *S*/*p* value of sows and the percentage of seronegative sows. Based on the median of the percentage of PUCS positive for PCV-2, the farms were classified in those with ≤ or >10% of samples positive. These two classes of farms were included as fixed effect in the model (PROC GLIMMIX) to identify as outcome the percentage of sows seronegative for PCV-2 in each farm (binomial distribution) and the percentage of farms with seronegative sows for PCV-2. In the last outcome variable, the farms were classified as those with the presence of at least one seronegative sow for PCV-2 (≥1 sow) and those with no seronegative sow (absence), and analyzed using binary distribution.

## 3 Results

**Figure 1:**
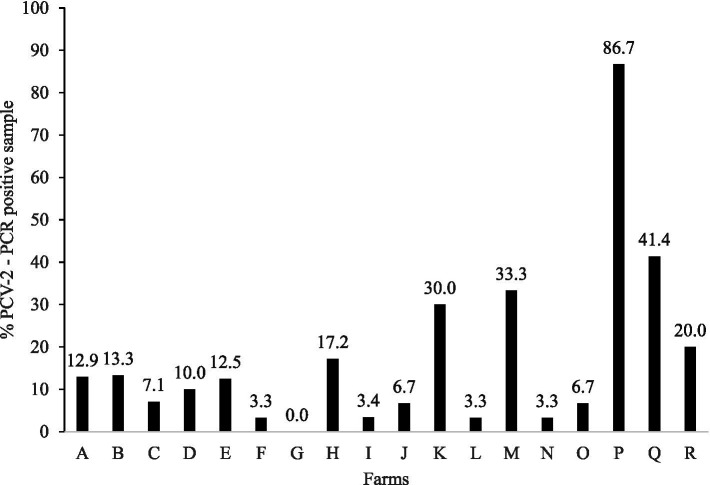
Frequency of PCV2-PCR positive placental umbilical cord serum (PUCS) from litters of 18 breeding farms.

**Figure 2:**
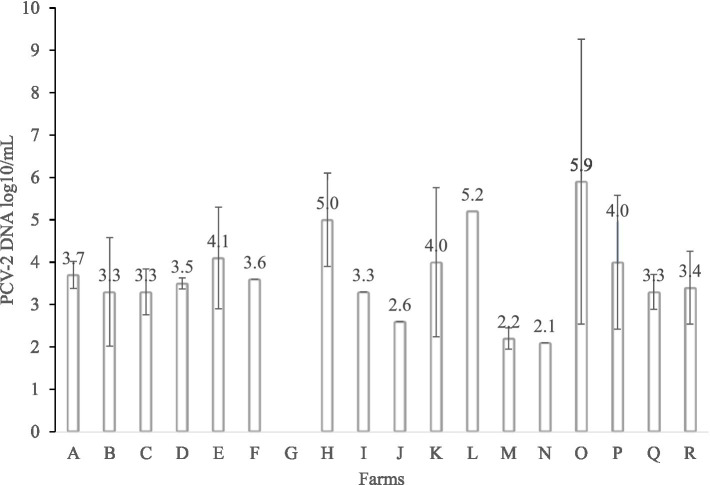
Average viral load of PCV2-positive PUCS samples with standard deviation in all 18 breeding farms. The results are expressed as the log10 viral copy number per milliliter of PUCS (copies/mL).

A total of 17.29% (93/538) of the PUCS showed positive (Ct < 37) for PCV-2 by qPCR (Figure 1). The average PCV-2 viral load was 10^3.6^ (lower quartile −10^3.3^; upper quartile −10^3.7^), and the farms O (10^5.9^ ± 7.3), L (10^5.2^), and H (10^4.9^ ± 1.1) had the highest PCV-2 viral load (Figure 2). The PCV-2 was detected in at least one PUCS in 94.4% of the sampled farms (17/18), with a frequency by farm ranging from 3.3 to 86.7% (Figure 1).

**Figure 3:**
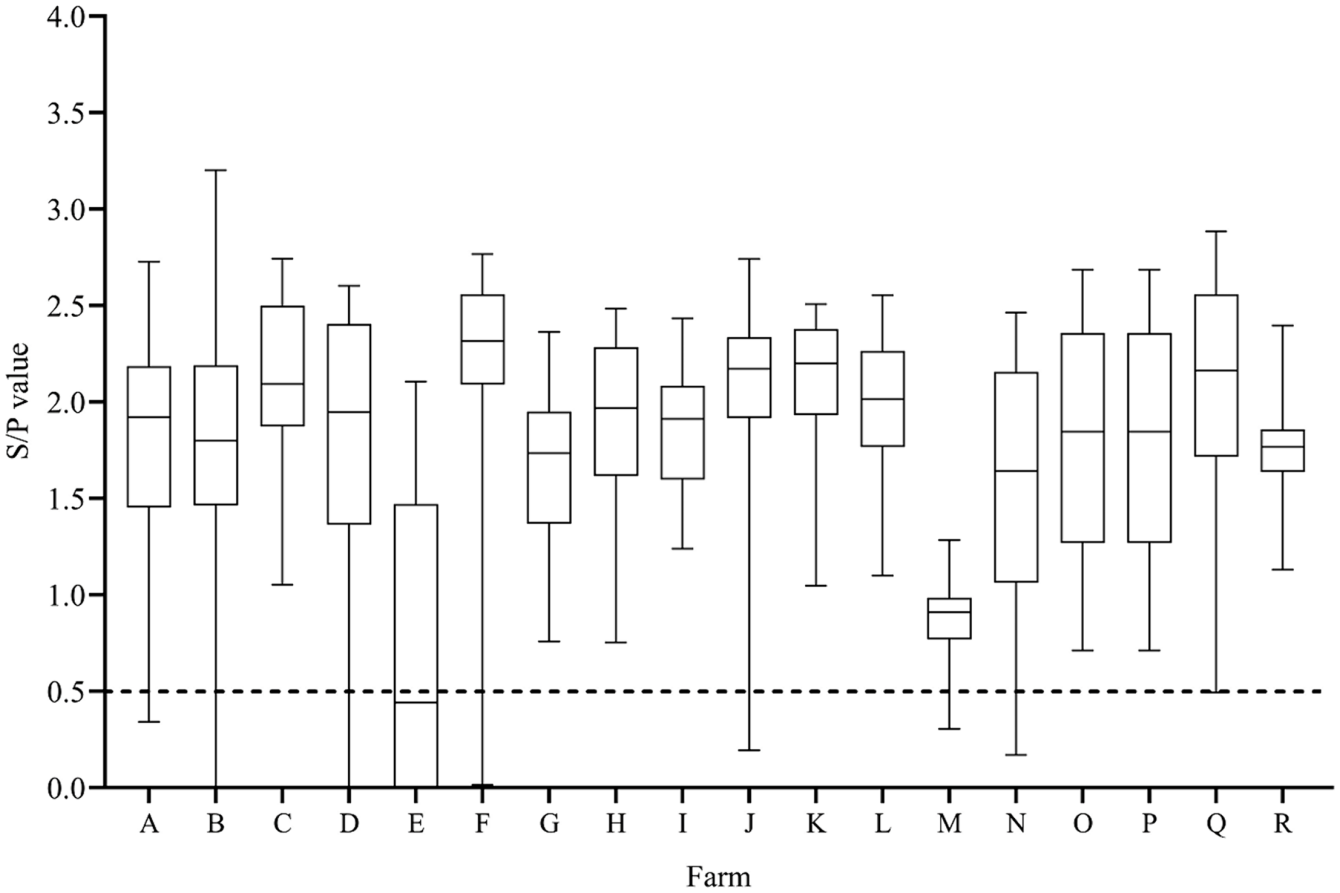
PCV-2 *S*/*p* values of farrowing sows (*n* = 538) in 18 farms. Sows were considered seropositive against PCV-2 when the *S*/*p* ratio was ≥0.5.

A total of 94.8% (510/538) sow serum samples showed positive antibodies for PCV-2. All 18 farms had at least 46.9% of PCV-2-seropositive sows. The average *S*/*p* value was 1.79 ± 0.03 (lower quartile −1.45; upper quartile −2.26), and farms P (2.23 ± 0.11) and F (2.27 ± 0.06) had the highest average serological values (Figure 3). Seronegative sows were detected in 8 out of the 18 farms (A – 1/31; B – 2/30; D – 2/30; E – 17/32; J – 1/30; M – 3/30, N – 1/30, and Q – 1/29).

There was no correlation (*p* ≥ 0.51) between the percentage of litters positive for PCV-2 by PUCS with the *S*/*p* value and the percentage of sows seronegative for PCV-2 in each farm (Table 2). However, a higher percentage of seronegative sows (*p* < 0.01) was observed in those farms with more than 10% of litters positive for PCV-2 by PCR (8.9 ± 1.7%) compared to those with ≤10% of PUCS-positive litters (1.5 ± 0.7%). In contrast, the frequency distribution of farms with at least one seronegative sow was not associated (*p* = 0.36) with the frequency of positive PUCS (≤ or >10%, Table 2).

**Table 2:** Association of farms (*n* = 18) with litters positive for PCV-2 by porcine umbilical cord serum (PUCS) and PCV-2-seronegative sows.

Correlation	*r*		*p*-value
Positive PUCS (%) vs Sows *S*/*p* value	−0.17		0.51
Positive PUCS (%) vs Seronegative sows (%)	−0.04		0.87
Frequencies	Positive PUCS (%)^†^		
	≤10	>10	
*n*	9	9	
Seronegative sows, %	1.5 ± 0.7	8.9 ± 1.7	<0.01
Farms with seronegative sows, %	33.3 ± 15.7	55.6 ± 16.6	0.36

*S*/*p* value, average of *S*/*p* value in each farm; Positive PUCS, percentage of litters positive for PCV-2 by PCR on each farm. Seronegative sows – percentage of sows seronegative for PCV-2 by ELISA on each farm. Farms with seronegative sows – farms with at least one sow seronegative for PCV-2. ^†^Farms with less or more than 10% of litters positive for PCV-2 by PCR, classified based on the median of the farms included in the study.

## 4 Discussion

The aim of this study was to assess the frequency of viremic newborn piglets for PCV-2 using qPCR from PUCS and to determine immune status of sows using serology (ELISA). Also, the relationship between seronegative sows and positive PUCS for PCV-2 in 18 breeding farms with no history of clinical PCVAD in Brazil was assessed.

To the best of the authors’ knowledge, this is the first study regarding PCV-2 detection in PUCS in Brazil.

Based on the results, 94.8% of the sows showed PCV-2 seropositivity at sampling, and high levels of PCV-2 antibodies were detected. Since the sows enrolled in this study were vaccinated only when they were around 21 days of age, the serological response detected by ELISA could have occurred just due infection because the duration of immunity of such vaccines are considered to last around 6 months (5). Despite the high frequency of seropositive samples (94.8%), seronegative sows were detected in 8 out of the 18 farms (44%). It was demonstrated that in multiplier farms, where only piglet vaccination is used, a part of these animals may be seronegative at the end of the fattening period (12). If some of these animals eventually become replacement stock, a percentage of seronegative gilts potentially becomes susceptible to infection, generating seronegative subpopulations (13). Taking this subpopulation of seronegative sows into consideration, sow vaccination should be considered to increase immunity and decrease virus circulation since the subclinical status can easily lead to a clinical PCVAD (22, 29).

Although a high proportion of sows presented antibodies against PCV-2, 94.4% of the farms presented at least one positive PUCS by PCR, with a frequency of 17.29%, reaching up to 86.67% in one of the farms. These data suggest the maintenance of the virus even in clinically stable farms (without a history of PCVAD in the last 12 months) with a high proportion of serologically positive sows. Studies performed in the USA with pre-suckling piglets showed a PCV-2 prevalence ranging from less than 10% (29) to up to 82% (15) by PCR, similar to our findings. In contrast, European studies reported no PCV-2 DNA detection in any of the pre-suckling piglets (14, 16). In one of these studies (14), only sow farms with less than 1,000 sows were selected. In our study, we selected farms with more than 1,000 sows, and farm size may have contributed to our results since the larger number of animals could have increased the odds of virus survival and circulation among pigs.

The presence of pre-suckling viremic piglets has been associated with a higher chance of horizontal transmission of PCV-2 among littermates, increasing the number of infected piglets at weaning, which also increases the risk of low vaccine efficacy (10). The higher risk of reducing vaccine efficacy occurs because piglets might already be infected with PCV-2 at the time of vaccination, and some of them may develop the clinical disease earlier or maintain a subclinical condition (PCV2-SI), which can impact the zootechnical performance (18–20). Some risk factors can also potentiate viral replication, facilitating the shift from a subclinical to a clinical condition. Some studies demonstrated that a nonspecific stimulation of the immune system, such as vaccination, combined with a preexistent subclinical and endemic PCV-2 infection, may favor the development of PCVAD (29, 30). Moreover, piglets are more likely to exhibit PMWS after early infection by PCV-2 and when weaned before 21 days (19). These risk factors are common in Brazilian farms, and the association of them with the presence of pre-suckling infected piglets may reduce the efficacy of PCV-2 piglet vaccination. We did not assess the piglets of this study during the nursery and/or finishing phases and could therefore not evaluate the impact of PCV-2 detection by PUCS on performance or clinical condition in the downstream flow. However, our results suggest that this should be investigated in future studies.

A higher percentage of seronegative sows (*p* < 0.01) was observed in those farms with more than 10% of litters positive for PCV-2 (8.9 ± 1.7%) compared to those with ≤10% of PUCS-positive litters (1.5 ± 0.7%). Most likely, the seronegative sows increase the frequency of infected newborn piglets (13–16), and colostrum protection from these sows to their offspring might be weakened (17).

Finally, the combined results of PUCS and serology suggest a heterogeneous immune situation of sow farms in Brazil. In this scenario, the assessment of PCV-2 infection in the early life of piglets would be important to clarify the infection dynamics in farms since the presence of pre-suckling infected piglets suggests a higher chance of the horizontal transmission of PCV-2 and/or an increased risk of low vaccine efficacy (10). The reduction in vaccination efficacy associated with risk factors can easily trigger PCVAD (19, 31, 32). In future studies, a longitudinal approach can be used to define the infection dynamic within the farms. Also, a more embracive study can be done with the correlation of reproductive data and PCV-2 infection dynamic. Another important pattern that could be analyzed is the impact of the prevalence within the sow farm and the impact of it in the following production stages. In conclusion, the results of our study indicate a high proportion of infected pigs showing no clinical signs on Brazilian farms, with newborn piglets detected positive for PCV-2, highlighting the need for preventive measures on these farms and on the downstream flow.

## Data availability statement

The datasets presented in this manuscript are not a vailable because access is subject to review by the data custodians. Requests to access the datasets should be directed to karinelt87@yahoo.com.br.

## Ethics statement

The animal study was approved by Ethics committee from Federal University of Rio Grande do Sul (UFRGS). The study was conducted in accordance with the local legislation and institutional requirements.

## Author contributions

RL: Writing – original draft. EC: Writing – original draft. LH: Writing – original draft. AT: Writing – original draft. FQ: Writing – original draft. AF: Writing – original draft. JP: Writing – original draft. RU: Writing – original draft. DB: Writing – original draft. KT: Writing – review & editing.
